# A TCR-switchable cell death pathway in T-ALL

**DOI:** 10.18632/oncoscience.342

**Published:** 2017-03-31

**Authors:** Christine Tran Quang, Benedetta Zaniboni, Jacques Ghysdael

**Affiliations:** Institut Curie, PSL Research University, CNRS UMR 3348, F-91405, Orsay, France; Université Paris Sud, Université Paris-Saclay, CNRS UMR 3348, F-91405 Orsay, France

**Keywords:** T-ALL, TCR signaling, cell death, negative selection, targeted therapy

The cell surface receptor for antigen in mature B (BCR) and T lymphocytes (TCR) is central to the adaptative immune response. Structurally, these receptors entails the association between clonotypic antigen binding chains (TCRα and TCRβ for the TCR), coupled to signaling chains (CD3ε,γ,δ and the ζ chains for the TCR). Emergence of many non-Hodgkin B cell lymphomas subtypes is commonly associated with antigenic BCR activation, activating mutations in BCR signaling chains and downstream adapters/effectors. Likewise, progression of certain T cell lymphomas is associated with gain-of function alterations in TCR signaling components. For example, Sezary syndrome cutaneous T cell lymphoma shows upregulation of the TCR LAT adaptor in most cases and frequent activating mutations in the adaptor CARD11 and in phospholipase Cγ1 [1].

**Figure 1:**
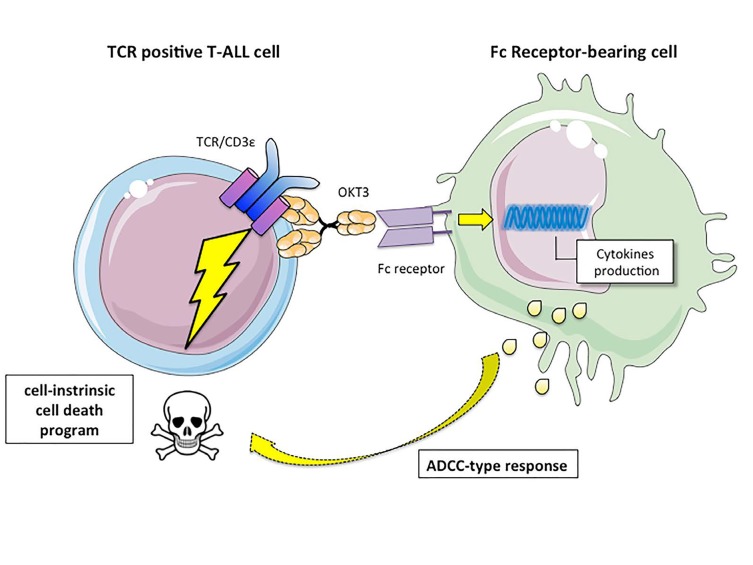
In T-ALL an intrinsic, TCR-induced cell death pathway activates leukemic cell apoptosis

T cell acute lymphoblastic leukemia (T-ALL) originates from the transformation of T cell progenitors, resulting in the accumulation of lymphoblasts arrested at specific stages of differentiation. T-ALL are classified into molecular subtypes characterized by abnormal expression of specific transcription factors (e.g. TAL1, LMO1/2, TLX1/3), involved in differentiation blockade. A number of additional genetic alterations are found across these subtypes, including activating mutations in NOTCH1 or the JAK/STAT pathway and inactivating mutations in several tumor suppressor genes [2]. TCR-expressing mature T-ALL represent about 50% of pediatric cases and 20% of adult cases. Whether TCR signaling contributes to leukemogenesis is unclear. Information gathered from T-ALL mouse models indicates that while signaling through the pre-TCR impinges upon leukemogenesis, the presence of a functional TCR is not critical [3-5]. TCR signaling is involved in a major developmental checkpoint during normal T cell development in the thymus. Thymocytes bearing a high affinity TCR for self-peptide/MHC complexes are deleted (negative selection) while those with a low affinity TCR survive and further differentiate into mature T cells (positive selection). We observed that co-expression of the TEL- JAK2 oncogene with a transgene encoding TCR-HY, which induces negative selection only in male mice, specifically compromised leukemia onset in males [4]. Importantly, in our new study [6], when leukemias obtained from females were transplanted in either male or female secondary recipients, only females succumbed to T-ALL. This indicated that the strong/sustained TCR activation associated with the negatively selecting TCR- HY severely impaired leukemia maintenance. Consistent with this, stimulation of a TCR-negative cell line engineered to express the TCR-HY transgene by the DBY cognate antigen resulted in dose-dependent cell death [6]. Modern multi-agent chemotherapy has considerably improved T-ALL outcome. However, about 15% pediatric and 40% adult patients relapse and overall survival is still below 25%, calling the search for alternative therapeutic approaches. As specific anti-TCR/CD3 antibodies can induce signaling, we investigated an immunotherapeutical approach using anti-CD3ε antibodies in T-ALL. *In vitro* treatment with anti-CD3ε specifically induced TCR signaling followed by apoptosis in 24/24 TCR-positive diagnostic T-ALL cases while sparing TCR-negative cases. Most importantly, *in vivo* expansion of 6/6 TCR-positive xenografts belonging to different T-ALL molecular subtypes was severely impaired by anti-CD3ε OKT3 mAb treatment, an anti-leukemic effect that translated into improved survival. OKT3 anti-leukemic effects can result from induction of a cell-intrinsic cell death program and/or antibody-dependent cell cytotoxicity (ADCC)-type responses. The fact that LAT expression knockdown in T-ALL strongly impaired the anti-leukemic response to OKT3 shows that, at least in NSG mice, OKT3-induced TCR signaling rather than ADCC-type responses is responsible for the anti-CD3 anti-leukemic effects [6]. Thus a latent cell death program, switchable by anti-CD3ε treatment, can be induced in T-ALL, which dominates the many distinct oncogenic pathways active in different tumors (Figure 1). We identified the transcriptional program associated with anti-CD3 treatment in T-ALL *in vitro* [6] and *in vivo* (our unpubl. obs.) and found it to resemble that of thymocyte negative selection, but to be distinct from that resulting from inactivation of T-ALL oncogenes [6,7]. Characterization of critical molecular effectors of this cell death program is ongoing and will allow identifying either synthetic lethal partners of anti- CD3 treatment or ways to bypass anti-CD3 itself to further improve the therapeutic potential of this pathway. This is important since our results show that the presence of TCR- negative subclones in otherwise TCR-positive T-ALL xenografts results in leukemia recurrence from OKT3- mediated therapy [6].

The selection of acquired mutations during T-ALL progression is associated with clonal evolution, resulting in coexistence at diagnosis of related clones endowed with distinct leukemogenic potential. Whether anti-CD3 treatment can impair the leukemia initiating (stem) potential of TCR-positive T-ALL remains to be investigated.

OKT3 has been used in the clinics since 1986 to treat allograft rejection. Its side effects in humans include strong immunogenicity and a cytokine-release (flu-like) syndrome. Since then, several humanized anti-CD3ε mAb further mutated in their Fc domain to impair FcR recognition have been developed [8]. Pre-clinical testing of these mAb for their anti-leukemic activity is ongoing in T-ALL xenografts. Irrespective of the fact that anti-CD3- based therapeutic approaches will or not prove useful in the clinic, our work in collaboration with the Asnafi laboratory [6] uncovered a conserved and switchable cell death pathway in T-ALL. Further dissection of this pathway to identify its intracellular effectors will provide alternatives to TCR-directed therapies that, in addition, might turn out to be relevant also to the treatment of TCR- negative T-ALL.
